# Relieving the Gambling Itch Through Alcohol Consumption: The Impact of COVID-19 Restrictions on Australian Casino Patrons

**DOI:** 10.1007/s10899-023-10252-9

**Published:** 2023-09-27

**Authors:** Tenghao Zhang, Pi-Shen Seet, Janice Redmond, Jalleh Sharafizad

**Affiliations:** 1 Australian Energy Market Operator, Perth, WA Australia; 2 School of Business and Law, Edith Cowan University, Joondalup, WA Australia

**Keywords:** COVID-19, Social distancing, Gambling, Alcohol consumption, Social network, Stress-response dampening

## Abstract

This paper extends our understanding of how casino patrons are affected by COVID-19 restrictions and how they cope by substituting gambling with alcohol consumption. We conducted two studies using a nationwide survey sample collected in Australia during the pandemic lockdown. Study 1 compares the casino patrons with two reference groups (other gambling patrons and non-gambling individuals) and investigates the lockdown restrictions on respondents’ relational strength, and their potential impact on mental health and future prospects. Study 2 applies the stress-response dampening model (SRD) and tests how respondents used alcohol consumption to cope with the lack of access to casinos during the lockdown. The results from Study 1 suggest that lockdown restrictions on respondents’ relational strength have significant negative impacts on anxiety, life satisfaction and post-pandemic outlook. Study 2 finds that casino patrons substituted gambling with alcohol consumption during the lockdown, with increased alcohol consumption negatively related to life satisfaction. Paradoxically, Australian gambling venue owners may not be adversely affected as many also run liquor retail operations.

## Introduction

The gambling sector relies heavily on its loyal customers (Prentice, 2013), as well as the gambling-based tourism and hospitality market. The unprecedented worldwide lockdown and social distancing restrictions in response to the COVID-19 pandemic has forced many casinos and various gambling venues to shut down indefinitely (Fernandes, 2020; Marsden et al., 2020). While casinos are struggling to survive the pandemic, *casino patrons* (i.e. those who gamble at casinos) may also suffer from social isolation and inaccessibility to their hobbies or even addictions (Hakansson et al., 2020).

Australia has one of the highest gambling participation rates in the world with about 39 percent of adults defined as “regular gamblers” with over one-fifth of these considered over-reliant on gambling (Armstrong & Carroll, 2017). Gambling taxation, on average, represents over 12 percent of Australian States’ and Territories’ taxation revenue (AGC, 2019), which makes the gambling industry critical in terms of supporting the wider post-pandemic economy recovery initiatives from a government perspective, and this is similar in other OECD countries like Italy (Raspor et al., 2019).

As one of the earliest Western countries affected by the pandemic, Australia responded with a prompt economic and travel lockdown with strict social distancing restrictions to stop the community spread of the virus. This has had enormous impacts on the economy and Australian residents (N. Biddle et al., 2020a, 2020b). Although no single industry could be spared from the impact of COVID-19, the hospitality industry has been arguably the most severely devastated by the pandemic, which is “affecting the DNA of hospitality at its core” (Rivera, 2020).

On 23 March 2020, Australia closed all casinos and gambling venues. This led to a sweeping and immediate impact on the gambling industry. For example, a major Australian casino gambling company laid off 90 percent of its staff in April (AAP, 2020). Although some casinos in Australia have subsequently re-opened with more limited offerings in some selected regions from June, the gambling industry is still deeply concerned whether their regular customers will come back (Asher, 2020). As international borders will remain closed in the foreseeable future, the heavily overseas customer-reliant Australian casinos (Forbes & Dyer, 2020) are in a crisis. Therefore, it is of vital importance for the gambling industry and all its stakeholders to understand how gambling patrons, especially those that go to casinos, are affected by the pandemic and what are their coping strategies.

Although there is some evidence showing a rise in online gambling (Han et al., 2019), online casinos and internet gambling are still illegal in Australia (Australian Government, 2001) and OECD countries like the USA, Norway and many members of the European Union (OECD, 2011). Various organizations have suggested that gambling and gambling-related problems could potentially rise during pandemics as people spend more time indoors, however, research in Australia has found that enforcing limitations on land-based gambling products during the COVID-19 pandemic did not lead to significant differences in the occurrence of gambling issues or online gambling behaviors in states that had restrictions as compared to those that did not have any (Black et al., 2022), the findings of the current study indicate otherwise. This differs from Sweden where the level of gambling activity decreased during the COVID-19 pandemic (Auer & Griffiths, 2022) and Canada where nearly one-third of gamblers reported stopping gambling altogether during lockdowns (Shaw et al., 2022). This suggests Australian customers or patrons may look for other legal means to help them relieve their gambling urges. Research has shown that this may take the form of increased alcohol consumption and/or other substance use (Barnes et al., 2005; Rice & Van Arsdale, 2010; Rueda Ruiz et al., 2023; Suomi et al., 2014; YalÇIn, 2023). However, the impact of these substitutions on gambling patrons has been relatively under-studied in the context of the COVID-19 casino closures.

To address this research gap, the main purpose of this study is to investigate (a) how casino patrons are affected by the pandemic restrictions and (b) how they cope with the impacts. Accordingly, we developed two sets of research questions:

1. *What are the impacts of the pandemic restrictions on casino patrons’ social networks and mental health? Are they different from other gambling patrons and non-gambling Australians?* Specially, from the social network perspective, we investigate how the government-imposed lockdown and social distancing restrictions affect casino patrons’ relational strengths with their close social networks and thereby influence their mental health such as anxiety and life satisfaction. Meanwhile, we are also interested in understanding how these factors affect their post-pandemic outlook. As the closures of casinos have forced casino patrons to stay away from their hobbies and addictions, we would thus expect they are likely to be different from other groups of individuals.
2. *How do casino patrons cope with the impact of the pandemic and their pre-existing addictive behaviors during the lockdown? Does alcohol consumption serve as an effective coping strategy?* If the analysis of the first research question suggests that COVID-19 restrictions have negative impacts on all casino patrons, then we will further restrict our samples to those with alcohol consumption habits. Drawing on stress-response dampening (SRD) (Levenson et al., 1980; Sher & Levenson, 1982), we speculate that during the pandemic lockdown, when almost all casinos and other gambling venues are closed (Fernandes, 2020), casino patrons tend to substitute their pre-existing addictive behaviors (gambling and alcohol consumption) with increased alcohol drinking as a strategy to cope with the pandemic. We also examine the effects of SRD on their subjective well-being during the lockdown.

To explore the questions above, we designed two studies to answer each research question, respectively. Study 1 entailed comparisons with two reference groups (other gambling patrons and non-gambling individuals), the gambling and alcohol-related variables will not be included in the model. Study 2 focused on the gambling and alcohol factors based on the SRD model and thus comparisons will be made between casino patrons and other gambling patrons.

Our results from Study 1 suggest that lockdown restrictions on respondents’ relational strength have significant negative impacts on anxiety, life satisfaction and post-pandemic outlook. In Study 2, we find that casino patrons substitute gambling with alcohol consumption during the lockdown and the increase of alcohol consumption was negatively related to life satisfaction.

Paradoxically, this supports the market findings that although the earnings of casinos may have been affected, it has not had a significant effect on the overall earnings of owners of casinos (Kang et al., 2011). This may be because many gambling venue operators in Australia also legally own alcohol retail outlets, which are important sources of revenue. Therefore, if gambling patrons increase their alcohol consumption, the companies may be less affected by the closure or restrictions on their casino operations.

The paper will next review the literature on our research questions and develop relevant hypotheses for each of the studies. This is followed by a description of the methodology. The data analysis and findings are then presented. The paper concludes with a discussion and main contributions to theory and practice as well as suggestions for future research.

## Study 1: Literature Review and Hypothesis Development

### Lockdown, Relational Strength, and Mental Health

Although it might be difficult to comprehensively test the applicability and validity of any well-established theories in the COVID-19 context in such a short period, preliminary empirical evidence suggests that the COVID-19 pandemic has a significant negative impact on individuals’ mental health (Armitage & Nellums, 2020; Dsouza et al., 2020; Zhang et al., 2020). In line with these studies, it is possible to gain an insight into this through the impact of the pandemic on casino patrons from a relational strength perspective.

During the COVID-19 pandemic, people may be affected by lockdown restrictions in terms of unmet belonging and social needs. Social network theory maintains that two types of social capital exist in individuals’ social networks: *bonding social capital* that occurs within a group such as family members and close friends, providing crucial help to group members who need it the most; and *bridging social capital* that helps information dissemination by connecting people from different groups such as business partners and distant acquaintances (Granovetter, 1977; Rost, 2011). Although opinions differ on the roles of the two types of social capital in individuals’ interpersonal relationships, the bonding relationship is usually more important in sustaining people’s daily lives and underpinning the bridging relationship (Rost, 2011). As such, this study will focus on bonding perspectives.

*Relational strength* refers to the strength of long-term bonding relationships between individuals (Song & Wang, 2013; Yang et al., 2019). It is reflected by the frequency and intensity of relationships (Tzabbar & Vestal, 2015), or the quality and reciprocal trust of relationships (Yang et al., 2019). In other words, relational strength signifies the accessibility and quality of a person’s bonding relationships. When the pandemic hit and resulted in nationwide lockdowns and social distancing restrictions, these inevitably affected peoples’ relational strength with their bonding networks.

There may be several scenarios of COVID-19 restrictions impacting on relational strength. On the one hand, as Günther‐Bel et al. (2020) report, the lockdown confines one’s household members such as children, spouse and parents together for a long period and this may improve, or deteriorate relationships. On the other hand, bonding networks include other non-household members, and any lockdown may negatively affect one’s networks with other family members and friends as people are isolated from regular social contacts and find it difficult to access necessary social help and acquire information (Armitage & Nellums, 2020). Collectively, the lockdown and social distancing restrictions would therefore most likely have a negative overall impact on individuals’ relational strength.

Hence, if there is a negative impact of pandemic restrictions on individuals’ relational strength, this would have an associated negative influence on people’s mental health. Studies have tested the relationship between bonding relationships and mental health in various settings. For example, Phongsavan et al. (2006) and De Silva et al. (2007) observe that individuals’ connections with their community are positively related to lower risk of mental distress. Lee’s (2013) study on social network websites indicates that the strength of bonding social capital is positively related to their low anxiety attachment. Economou et al. (2014) find that during the Greek economic crisis when there was an unusually high unemployment rate, individuals’ social capital level was negatively related to depression and anxiety. Accordingly, when individuals’ relational strength is affected by a force majeure event like the pandemic lockdown, it is likely to give rise to anxiety and loneliness (Pattison et al., 1979), as well as decrease of life satisfaction (Zhang et al., 2020).

For casino and other gambling patrons, the impact of pandemic lockdowns can be significant. Most gambling venues have been closed (Fernandes, 2020; Marsden et al., 2020), and many online gambling games like horse betting and some lottery games such as sports betting are also restricted due to cancellations of various public gathering events. Furthermore, many informal gambling games like cards and mahjong which are often played within close-knit relationships like close friends and relatives are also unable to proceed under the social distancing rules. Previous studies have suggested that many gambling players use gambling as a means of social networking (Meisel et al., 2013) and some of them even begin gambling based on their social networks (Reith & Dobbie, 2011). Consequently, the lockdown impacts on these people can be considerable.

Taken together, it is suggested that the greater impact of social distancing and lockdown restrictions on relational strength of the casino patrons, the higher the anxiety level of these people, and consequently this will lead to a decrease in their life satisfaction and a rise in other related stresses during the lockdown period (Price, 2022). Meanwhile, we also postulate that relational strength can directly affect life satisfaction, and that compared with other gambling patrons and non-gambling individuals, relational strength impact has higher negative relationship with life satisfaction, higher positive relationship with anxiety, while anxiety also has a higher negative relationship with life satisfaction. Thus, we propose the following hypotheses during the COVID-19 pandemic lockdown period among casino patrons:

#### Hypothesis 1

Relational strength impact is negatively related to life satisfaction.

#### Hypothesis 2

Anxiety during the lockdown period is negatively related to life satisfaction.

#### Hypothesis 3

Relational strength impact is positively related to anxiety.

### Relational Strength, Mental Health and Post-Pandemic Outlook

Understanding individuals’ prospect of the post-pandemic world is important for the gambling industry to help it prepare better for its reopening and also for policymakers in terms of comprehending its economic consequences. If people are optimistic about the post-pandemic trajectory, then the economy is more likely to usher in a V-shaped recovery (Best, 2020). Some early studies (e.g., Li et al., 2020) have found that COVID-19 has significantly changed tourists’ short-term post-pandemic travelling intentions. However, little is known about the long-term outlook. Although reopening casinos may give some relief to the gambling industry, operators and practitioners will need to be concerned about whether their patrons will become more pessimistic or more optimistic about the post-pandemic outlook and if their gambling habits have changed (Asher, 2020).

Previous research suggests a positive relationship between current life satisfaction and future outlook/planning (Azizli et al., 2015; Oishi et al., 2000). Meanwhile, direct relationships between individuals’ current social capital/network (Oshri et al., 2018) and anxiety (Rajandram et al., 2011) with future outlook have also been found in existing research. In the COVID-19 context, although individuals’ mental health and relational strength impacts may be temporary, we expect that these can still significantly affect post-pandemic outlook. Therefore, the following hypotheses are relevant among casino patrons:

#### Hypothesis 4

Anxiety is negatively related to post-pandemic outlook.

#### Hypothesis 5

Relational strength impact is negatively related to post-pandemic outlook.

#### Hypothesis 6

Life satisfaction during the pandemic is positively related to post-pandemic outlook.

### The Mediating Role of Life Satisfaction with Post-Pandemic Outlook

**Fig. 1:**
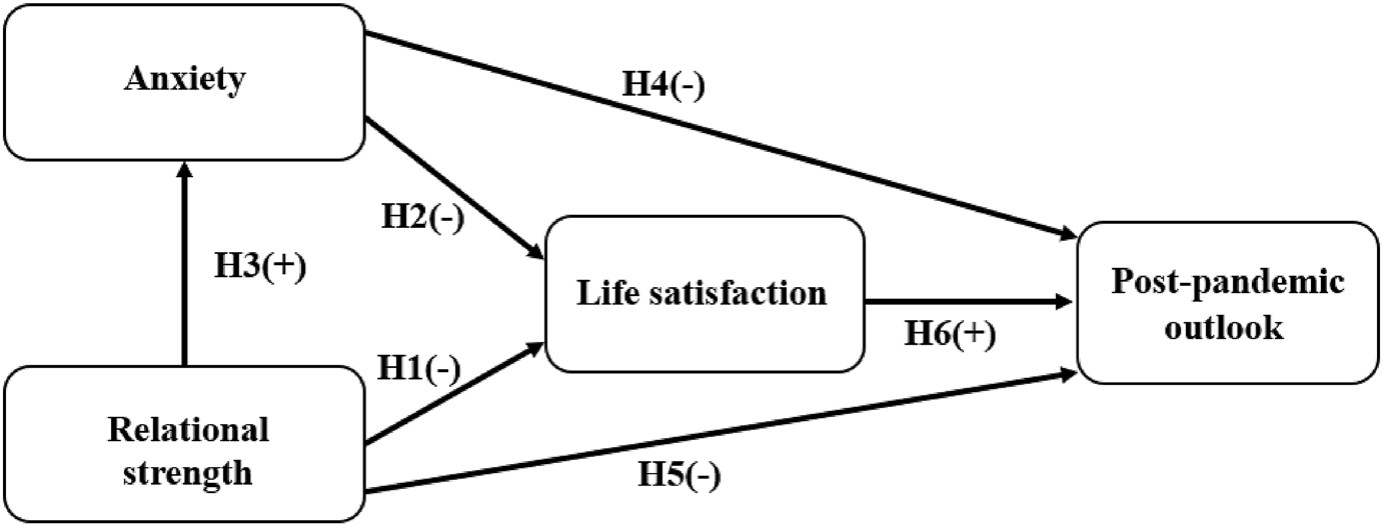
Conceptual model for Study 1

Given our earlier hypotheses (see Fig. 1) and discussion, life satisfaction may also mediate anxiety and relational strength with post-pandemic outlook. Thus, the following two mediation hypotheses are proposed for casino patrons:

#### Hypothesis 7a

Life satisfaction mediates the link of anxiety with post-pandemic outlook.

#### Hypothesis 7b

Life satisfaction mediates the link of relational strength with post-pandemic outlook.

## Study 2: Literature Review and Hypothesis Development

### External Stressors-Based Alcohol Consumption

Alcohol consumption has been frequently associated with stress (Clay & Parker, 2020; Hight & Park, 2019; Suomi et al., 2014). The stress-response dampening (SRD) effects of alcohol (Levenson et al., 1980; Sher & Levenson, 1982) has been extensively examined in alcohol consumption research (Armeli et al., 2003; Backer-Fulghum et al., 2012; Hight & Park, 2019; Sher et al., 2007). The SRD model suggests that certain individuals drink alcohol to escape from negative experiences and alleviate stress. Alcohol consumption as a “negative reinforcer” (Backer-Fulghum et al., 2012) motivates people to drink when they encounter, or are about to encounter stressful experiences. According to SRD research, the SRD process can be partitioned into two phases: *Phase One* is coping with stressors by drinking alcohol, and *Phase Two* involves the effects of alcohol consumption, i.e. whether it reduced the negative mood and stress or not (Levenson et al., 1980; Sher & Levenson, 1982; Sher et al., 2007).

Regarding Phase One, Cooper (1994) proposes the term “drinking to cope” (DTC) to explain why certain individuals prefer to drink alcohol to deal with stressors. Individuals with high DTC are more likely to increase their alcohol consumption when under stressful situations (Hussong et al., 2005; Rice & Van Arsdale, 2010). DTC involves a series of social and psychosocial motivations that give rise to alcohol consumption as a stress-coping strategy (Cooper, 1994). One of the most salient indicators of SRD alcohol drinking motivations is the individual’s alcohol drinking dependence, in other words, how often does the individual drink in normal times in the absence of crisis. Individuals with a higher alcohol dependence or positive drinking history are more likely to use DTC to deal with stress (Armeli et al., 2003; Levenson et al., 1987).

The COVID-19 pandemic can be regarded as a major external stressor (Price et al., 2022). Due to the major negative effects of the pandemic to the economy (Fernandes, 2020), employment (Zhang et al., 2020) and mental health (Dsouza et al., 2020), the pandemic effects are distinct from other types of stressors that have been discussed in previous SRD studies. Therefore, we anticipate that individuals in this study will also increase their alcohol consumption, and the incremental extent is predicated on individuals’ alcohol dependence. Thus, in the context of the COVID-19 restrictions, we propose the following hypothesis among casino patrons:

#### Hypothesis 8

Alcohol dependence is positively related to change in alcohol consumption.

Research has also shown that gambling shares a relationship with alcohol consumption (Barnes et al., 2009; Suomi et al., 2014). As such, for Study 2, we restrict the respondents to those casino patrons who have both recent gambling and alcohol consumption experiences. Hence, during the lockdown, when most gambling venues are unavailable, but alcohol is still easily accessible, we assume some casino patrons might substitute gambling with alcohol. From the SRD perspective, when their gambling needs cannot be fulfilled due to the pandemic confinement, it can become a stressor and make them anxious (Hakansson et al., 2020). Therefore, they might resort to alcohol consumption as a DTC strategy, i.e. those with a higher gambling dependence tend to increase the extent of their alcohol consumption during the lockdown. Hence, for casino patrons:

#### Hypothesis 9

Gambling dependence is positively related to their change in alcohol consumption.

### The Effects of “Drinking to Cope”

Pertaining to Phase Two of the SRD model, prior research has produces rather mixed findings on whether alcohol drinking, as a stress-coping strategy, actually alleviates stress. Although individuals’ DTC primarily focuses on stress reduction (Cooper, 1994), the actual SRD effects vary depending on situational and individual factors (Hight & Park, 2019; Sayette, 1999), and there is little consensus as to whether or under what circumstances alcohol consumption reduces stress (Sher et al., 2007). For example, Rice and Van Arsdale (2010) find significant positive relationships between stress, drinking to cope, and alcohol-related problems (i.e. stress indirectly leads to alcohol-related problems through the mediating role of DCT strategies). Backer-Fulghum et al. (2012) suggest that negative parental bonds are linked to an increase in alcohol consumption and result in more alcohol-related problems. Sinha et al. (1998), however, do not find positive SRD results among male participants with high drinking dependence but other research has found that heavy alcohol use was strongly associated with regular gambling during lockdown among young adults in the UK (Emond et al., 2022).

Existing research suggests that positive SRD results, such as stress reduction after drinking alcohol, mostly occur in regular or moderate alcohol dependence individuals (Giousmpasoglou et al., 2018; Hight & Park, 2019; Sher et al., 2007). In contrast, negative SRD results (e.g. increased stress and/or increase alcohol-related problems), are mostly found in individuals with high alcohol dependence or other long-standing problems (Backer-Fulghum et al., 2012; Rice & Van Arsdale, 2010).

Considering the continuing and unpredictable effects of the pandemic as a major external stressor, we postulate a negative relationship between increased alcohol consumption and life satisfaction:

#### Hypothesis 10

Change in alcohol consumption is negatively related to life satisfaction.

**Fig. 2:**
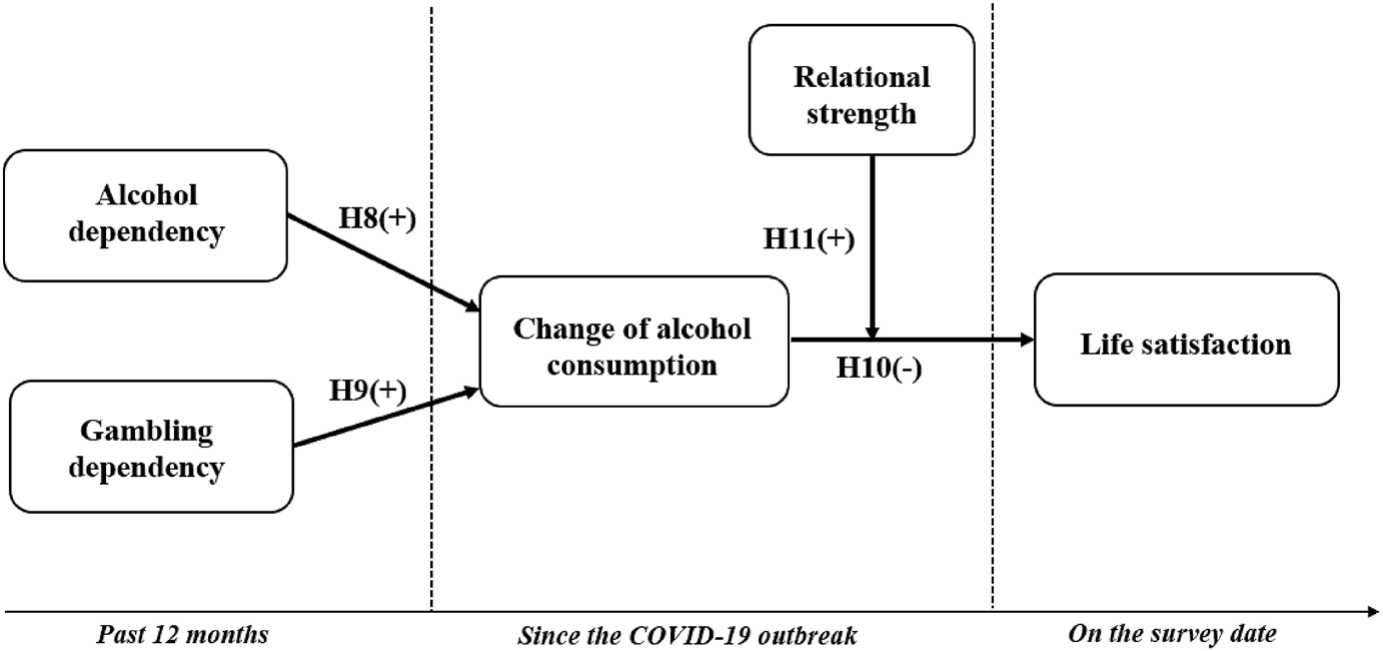
Conceptual model for Study 2

Given the strong causal power of relational strength impact on other variables that we hypothesized in Study 1, we would expect it to play a moderating role in this SRD-based model. Specifically, the negative relationship between change in alcohol consumption and life satisfaction is stronger in those whose relational strength is more negatively impacted by lockdown restrictions. Hence, during COVID-19 restrictions, we propose that (Fig. 2):

#### Hypothesis 11

Relational strength impact positively moderates the relationship between change in alcohol consumption and life satisfaction.

## Methodology

### Sample and Data

Data for the empirical analysis was extracted from the 34th ANU Poll (Nicholas Biddle et al., 2020a, 2020b), a nationally representative survey collected using Life in Australia™, the country’s only probability-based sample (N. Biddle et al., 2020a, 2020b). The most recent poll was part of the “COVID-19 attitudes and behaviours” monitoring surveys (N. Biddle et al., 2020a, 2020b) collected over the period 11th and 25th of May 2020, with a new section (substance use) and a new module (gambling) added to the questionnaire, which facilitates a deeper understanding of how Australians were coping with the pandemic. A total of 3930 individuals were invited to participate in the survey and 3249 had completed the questionnaire (82.7%). Nineteen respondents with significant missing values were excluded and this resulted in a total of 3230 samples.

This study focuses on casino patrons, although we also compare them with others. In Study 1, we partitioned the samples into three groups: casino patrons, other gambling patrons and non-gambling individuals. For casino patrons, the sample was restricted to those who had recent casino gambling experiences. The “gambling” module in the survey included multiple-choice questions asking the respondents to tick which of the listed 11 different gambling types they had played during the last twelve months. Among the 11 gambling types, two were casino-related (including play at a casino or similar venues), and nine were other types such as horse betting and participating in raffles. We restricted the samples to those who selected at least one casino gambling choice, which resulted in 331 samples. Then we selected those who chose other gambling types but did not select the casino gambling options (N = 1424). The remaining sample constituted the non-gambling individuals (N = 1475). In Study 2, the samples were further restricted to those who had both recent gambling and alcohol consumption habits. This was done using a question in the “substance use” section that asked respondents how often they drank alcohol in the last twelve months, and we excluded those who selected “do not drink alcohol,” which resulted in 292 samples for casino patrons and 1229 samples for other gambling patrons.

### Measures

To assess reliability, we used internal consistency (Cronbach’s α) for the two multi-item measures (anxiety and relational strength) which are reported below and are both within the acceptable range (α > 0.7) (Taber, 2018). For single-item measures such as life satisfaction and alcohol dependence, traditional reliability analysis like Cronbach’s α is less applicable (Dolbier et al., 2005). To deal with this, we conducted model validity analysis (see Sect. "Model Validity" and Table 3).

#### Anxiety

Many scales have been proposed to measure anxiety in the extant literature, however, none of them has been fully tested in the current pandemic context. This study focuses on individuals’ anxiety during the lockdown, therefore, six five-point point Likert scale items, derived from the widely used K6 scale (Kessler et al., 2002), were adopted which ask the respondents: “In the past four weeks, how you have been feeling: 1.nervous, 2.hopeless, 3.restless or fidgety, 4.everything was an effort, 5.nothing could cheer me up, 6.worthless (1 = none of the time, 5 = all of the time, Cronbach’s α = 0.88)”. The higher the score, the more anxious the respondent is. It should be noted that “past four weeks” in the question refers to April to May 2020, which was the strictest lockdown period in Australia since the outbreak.

#### Relational Strength

Two five-point point Likert items were used to measure the pandemic impact on relational strength: (1) “Since the spread of COVID-19 in Australia, how easy has it been to stay connected with family/friends outside your household?” (1 = very easy, 5 = very hard) and (2) “Since the spread of COVID-19 in Australia, how has the quality of your relationships with other people/family members in your household changed?” (1 = a lot closer, 5 = a lot more difficult). Cronbach’s α = 0.71. The two items were partially adapted from previous studies on relational strength (Song & Wang, 2013; Tzabbar & Vestal, 2015) with COVID-19 factors incorporated. The first question addresses individuals’ accessibility to relationships with their bonding social networks outside the household, while the second item asks about the quality of relationship within one’s household. The higher the score, the more negative the impact of the pandemic on relational strength.

#### Life Satisfaction

Following several previous studies (Boehm et al., 2015; Manning et al., 2016), the survey used a 0–10 scale to measure respondents’ general perception of their present life: “Overall, how satisfied are you with life as a whole these days?” (0 = not at all satisfied, 10 = completely satisfied). A higher score indicates greater life satisfaction during the pandemic.

#### Post-Pandemic Outlook

This survey used a five-point Likert scale asking respondents about their perceptions about the long-term future: “How has your outlook for your longer-term future (5–10 years from now) changed since the spread of COVID-19?” (1 = I feel a lot more negative, 5 = I feel a lot more positive). Considering that the COVID-19 impact on the global economy may last for several years, we believe that asking respondents about their long term rather than short term prospects is more appropriate.

#### Gambling Dependence

We aggregated the 11 dichotomous questions in the *gambling* module which ask the respondents to identify the gambling games (such as playing slot machines and Keno) that they had played for money in the last 12 months (Nicholas Biddle et al., 2020a, 2020b). The higher the score, the more dependent the respondents were on gambling.

#### Alcohol Dependence

A seven-point scale question was used to quantify respondents’ frequency of alcohol drinking in the last year (1 = less often than one day a month, 7 = every day) (Nicholas Biddle et al., 2020a, 2020b).

#### Change in Alcohol Consumption

A five-point Likert scale asked: “Since the spread of COVID-19 in Australia, are you drinking more or less alcohol?” (1 = a lot less, 5 = a lot more) (Nicholas Biddle et al., 2020a, 2020b). We reversed the original scoring such that the higher the score, the higher the alcohol consumption compared with the period before the pandemic.

#### Control Variables

We controlled for some factors that can affect the dependent variables. Age (Steinberg et al., 2009) and education (Webley & Nyhus, 2006) were controlled in Study 1 as they were found to influence people’s future outlook/orientation in the literature. Gender was controlled for in Study 2 as some studies found significant gender differences in SRD effects (Armeli et al., 2003; Sinha et al., 1998). While this study focusses on casino patrons, as the lockdown affected individuals differently depending on whether they gambled in casinos, other venues or did not gamble, we also anticipate that there are group differences between casino patrons, other gambling patrons and nongambling individuals. Therefore, we controlled for group differences in both studies i.e. all the three groups that were controlled for in Study 1, casino patrons and other gambling patrons were also controlled for in Study 2.

### Analysis Method

Structural equation modelling (SEM) with a Bayesian estimator was conducted in *Mplus* 7.4 (Muthén, 2010) to test the two models. Different from the frequentist analysis such as Maximum Likelihood Estimation (MLE) or resampling technique like the Bootstrapping process, Bayesian estimation combines prior distributions for parameters with data likelihood to form the *posterior distributions* for the parameter estimates, and posterior distributions are then applied into the analysis to estimate the probability of a hypothesized model (Besson et al., 2007; Mahoney et al., 2014). Although not without its critics, a growing number of studies have shown that the Bayesian approach has the ability to handle more complex models, provides more accurate estimates when the sample is small, and multicollinearity poses less of a problem (Muthén, 2010; Simon et al., 2015; Zyphur & Oswald, 2015).

## Results

### Descriptive Statistics

The demographic profiles of the three groups of respondents are reported in Table 1. The casino patron group’s correlation matrix is presented in Table 2a and. Overall, the two gambling patron groups are older and less educated than the non-gambling group. Meanwhile, clear gradient differences in terms of gender composition and prevalence of alcohol drinking can be noticed between the three groups: the casino patron group had the highest male proportion and alcohol drinking rate, followed by other gambling patrons, and then non-gambling group.

**Table 1:** Demographic profiles of respondents

Description		Casino patrons N = 331		Other gambling patrons N = 1424		Non-gambling group N = 1475	
		N	%	N	%	N	%
Age group	18–29	32	9.7	74	5.2	201	13.6
	30–49	80	24.2	400	28.1	522	35.4
	50–64	116	35.0	464	32.6	355	24.1
	65 and more	103	31.1	486	34.1	397	26.9
Gender	Female	162	48.9	779	54.7	874	59.3
	Male	169	51.1	645	45.3	601	40.7
Education	Secondary school or less	120	36.3	388	27.3	322	21.8
	Diploma or equivalent	114	34.4	439	30.8	360	24.4
	Bachelor or higher	97	29.3	597	41.9	793	53.8
Drinking habit	Alcohol drinker	292	88.2	1229	86.3	1164	78.9
	Non-drinker	39	11.8	195	13.7	311	21.1

There were a few missing values in gender and education variables, we replaced them using the mean substitution method

**Table 2:** Means, SDs, reliability scores and correlations among study variables

	Variables	Mean	SD	Skew	Kurt	1	2	3
*a: Casino patrons (Study 1)*								
1	Strength	5.42	1.36	.41	.28			
2	Anxiety	11.00	4.35	.98	.79	.31**		
3	Life	6.88	1.86	− 1.03	1.69	− .25**	− .49**	
4	Outlook	2.67	.86	.29	.13	− .28**	− .16**	.29**

	Variables	Mean	SD	Skew	Kurt	1	2	3	4
b: Casino patrons (Study 2)									
1	Alco. depend	4.03	1.78	− .11	− .86				
2	Gamb. depend	3.53	1.75	.78	.64	.06			
3	CAC	2.92	.96	− .42	− .08	.43**	.16**		
4	Strength	5.42	1.35	.42	.27	.03	− .02	− .06	
5	Life	6.87	1.89	− 1.07	1.73	.01	.03	.15**	− .25**

N = 332 (a); N = 291 (b). Skew. = Skewness; Kurt = Kurtosis; Strength = relational strength; CAC = change in alcohol consumption; Alco. depend = alcohol dependence; Gamb. depend = Gambling dependence; Life = life satisfaction; Outlook = post-pandemic outlook; **P < .01, *P < .05 (two-tailed)

### Model Validity

Following Muthén and Asparouhov (2012) and Zyphur and Oswald (2015), we adopted two measures to test model validity: model convergence and model fit. Model convergence test is carried out using an iterative process called MCMC estimation. Convergence is evaluated by calculating the potential scale reduction (PSR) (Gill, 2014). A PSR value not much larger than 1 is considered good model convergence, while Muthén and Asparouhov (2012) suggested below 1.1 to be considered as the threshold of good convergence. Model fit assessment is conducted using posterior predictive checking (Zyphur & Oswald, 2015), which computes a posterior predictive *p*-value (PPP). The PPP value is similar to an SEM fit index which compares the deviation degree between the observed data and generated data (Mahoney et al., 2014). A PPP value around 0.5 implies a good model fit, while > 0.01 or > 0.05 are all considered acceptable (Muthén & Asparouhov, 2012; Zyphur & Oswald, 2015).

### Parameter Estimation Results of Study 1

Table 3a and b show the results of parameter estimates for Study 1.

**Table 3:** Parameter estimations of Study 1

Parameter	Model 1				Model 2				Model 3			
	SE	SD	*P*	95% CI	SE	SD	*P*	95% CI	SE	SD	*P*	95% CI
a. Direct parameter estimations of Study 1												
Anxiety → Life	− .46	.05	.00	[− .57, − .36]*	− .50	.02	.00	[− .55, − .45]*	− .47	.02	.00	[− .51, − .42]*
Strength → Life	− .10	.05	.05	[− .20, − .00]*	− .13	.02	.00	[− .17, − .08]*	− .11	.02	.00	[− .16, − .06]*
Strength → Anxiety	.30	.06	.00	[.20, .41]*	.26	.03	.00	[.21, .31]*	.29	.03	.00	[.24, .34]*
Life → Outlook	.25	.07	.00	[.11, .38]*	.17	.03	.00	[.10, .24]*	.28	.03	.00	[.21, .34]*
Anxiety → Outlook	.04	.06	.25	[− .07, .16]	− .08	.03	.00	[− .14, − .02]*	− .02	.03	.32	[− .07, .04]
Strength → Outlook	− .23	.06	.00	[− .33, − .12]*	− .19	.03	.00	[− .24, − .14]*	− .17	.03	.00	[− .22, − .12]*

Mediator and indirect paths	Model 1				Model 2				Model 3			
	SE	SD	*P*	95% CI	SE	SD	*P*	95% CI	SE	SD	*P*	95% CI
b. Indirect parameter estimations of Study 1												
*Life*												
Anxiety → Outlook	− .12	.03	.00	[− .18, − .05]*	− .09	.02	.00	[− .12, − .05]*	− .13	.02	.00	[− .16, − .10]*
Strength → Outlook	− .03	.02	.05	[− .06, − .00]*	− .02	.01	.00	[− .03, − .01]*	− .03	.01	.00	[− .05, − .02]*
*Anxiety*												
Strength → Life	− .14	.03	.00	[− .20, − .10]*	− .13	.01	.00	[− .16, − .11]*	− .13	.01	.00	[− .16, − .11]*

Model 1: casino patrons (N = 331); Model 2: other gambling patrons (N = 1424); Model 3: non-gambling group (N = 1475). Model validity: PSR < 1.05, PPP > .05. SE: standard estimate. SD: standard deviation. 95% CI means the lower 2.5% and upper 2.5% at 95% credibility interval. Asterisk * suggests the 95% CI does not encompass 0. Variables’ full names see the note underneath Table 2. *P*-value: one-tailed

Regarding the three control variables, we found age and education were not significantly related to our model. As there were some noticeable group differences between the groups, three models are presented below for the three groups.

In Model 1, among casino patrons, all the direct paths except the *anxiety → outlook* relationship were significant. Thus, hypotheses 1, 2, 3, 5, 6 were supported, while Hypothesis 4 was rejected. Anxiety had the most negative correlation with life satisfaction (β = − 0.46, *p* < 0.01). Relational strength impact had a relatively small but significant negative relationship with life satisfaction (β = − 0.10, *p* < 0.05). However, it had a stronger influence on the post-pandemic outlook (β = − 0.23, *p* < 0.01). Relational strength was positively associated with anxiety (β = 0.30, *p* < 0.01).

To test the mediating effects of life satisfaction on post-pandemic outlook, we used the *Model Constraint* command in Mplus (Simon et al., 2015). The indirect effect size of the predictor anxiety on outlook through life satisfaction was significant (β = − 0.12, *p* < 0.01). Relational strength on life satisfaction through anxiety (β = − 0.14, *p* < 0.01) was also significant, as well as relational strength on outlook via life satisfaction, albeit with a relatively small effect size (β = − 0.03, *p* < 0.05). Meanwhile, if we combine both the direct and indirect effects, the total effect of relational strength on life satisfaction (β = − 0.24, p < 0.05) is similar to the *relational strength → outlook* link. Accordingly, Hypothesis 7a and 7b were supported.

Comparisons between casino patrons and other two groups are shown in Model 2 and Model 3 of Table 3. Overall, most parameter estimations were roughly consistent across the three models but there were some discrepancies. For example, the *anxiety → outlook* path was significant among other non-casino gambling patrons group (β = − 0.08, *p* < 0.01). The effect size of relational strength on outlook was substantially smaller among the non-gambling group (β = − 0.17, *p* < 0.01) than casino patrons. This suggests that the pandemic restrictions had a more negative impact in terms of post-pandemic expectations among casino patrons than the other two groups.

### Parameter Estimation Results of Study 2

The results of parameter estimates for Study 2 are presented in Table 4.

**Table 4:** Parameter estimations of Study 2

Parameter	Model A				Model B			
	SE	SD	*P*	95% CI	SE	SD	*P*	95% CI
Gamb. depend. → CAC	.13	.05	.02	[.01, .22]*	.01	.03	.40	[− .06, .06]
Alco. depend. → CAC	.41	.05	.00	[.28, .53]*	.36	.03	.00	[.29, .41]*
CAC → Life	− .16	.06	.00	[− .28, .03]*	− .04	.03	.06	[− .11, .02]
*Moderating effect*								
Strength × CAC → Life	.09	.06	.05	[+ .00, .17]*	− .02	.03	.23	[− .08, .01]
*Indirect paths (through CAC)*								
Gamb.depend. → Life	− .02	.01	.02	[− .05, − .01]*	.00	.02	.41	[− .00, .00]
Alco.depend. → Life	− .06	.03	.00	[− .11, − .01]*	− .02	.01	.07	[− .04, .00]

Model 1: casino patrons with alcohol drinking habits (N = 292); Model 2: other gambling patrons with alcohol drinking habits (N = 1229); Model validity: PSR < 1.05, PPP > .05. Variables’ full names see the note underneath Table 2. *P*-value: one-tailed

The three direct effects were all significant in Model A, hence, Hypothesis 8, 9, 10 were supported. Alcohol dependence had a strong predictive power on change in alcohol consumption (CAC) (β = 0.41, *p* < 0.01), which supports the SRD effect that people with higher alcohol dependence are more likely to use DTC when under stress. Gambling dependence also positively predicts CAC (β = 0.13, *p* < 0.05), This confirms our expectation that people with higher gambling dependence are more likely to substitute gambling with alcohol when gambling is unavailable. The negative CAC → life satisfaction correlation (β = − 0.16, *p* < 0.01) suggests that DTC did not necessarily lead to stress reduction.

Regarding the moderating effects of relational strength, we measured the interaction effect between the moderator (relational strength) and the predictor (CAC). The results indicate that relational strength positively moderates the CAC* → *life satisfaction path (β = 0.09, *p* < 0.05), and the effect size was stronger among the male cohort (β = 0.14, *p* < 0.05). Hence, Hypothesis 11 was supported. Additionally, we also found that alcohol and gambling dependencies can indirectly affect life satisfaction via the mediating effect of CAC, as the two indirect paths were all statistically significant.

**Fig. 3:**
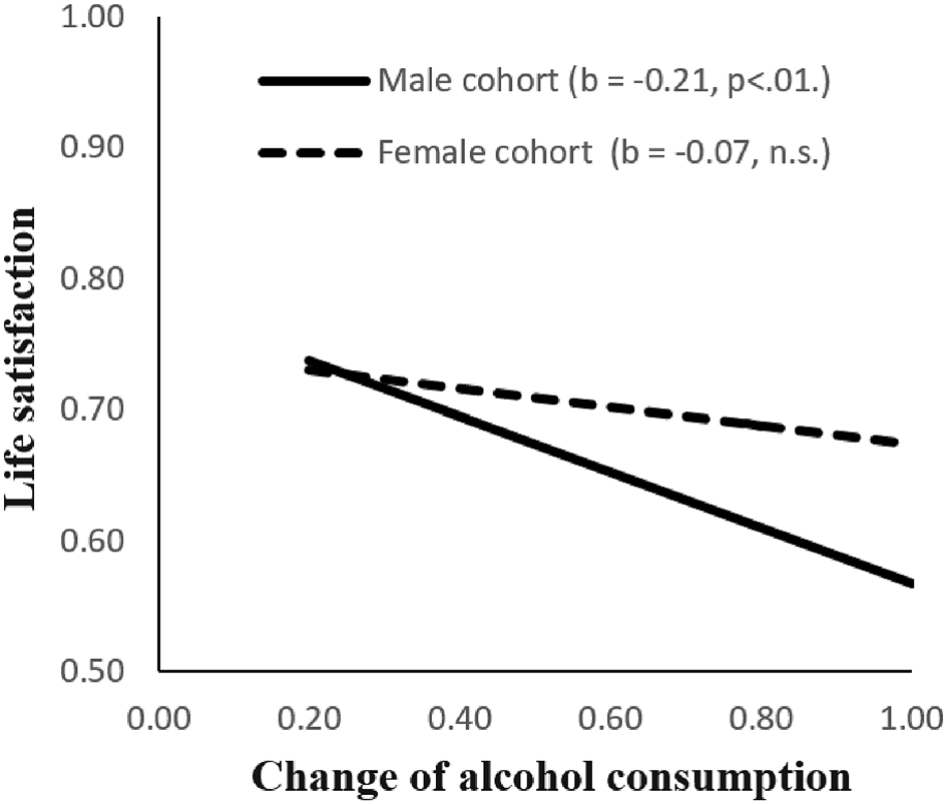
Gender differences in change in alcohol consumption and life satisfaction links. *Note* Male casino patrons: N = 156; Female casino patrons: N = 136

As for the two control variables, Fig. 3 shows comparison of the CAC* → *life satisfaction relationship between male and female casino patrons. It suggests that the effect size was more significant in men (β = − 0.21, *p* < 0.01) compared with women (β = − 0.07, n.s.).

The comparison between casino patrons with other gambling patrons is presented in Model B of Table 4. The results were surprising: in Model B, only the alcohol dependence → CAC relationship was significant (β = 0.36, *p* < 0.01). All other parameters were not significant, which means the conceptual model for Study 2 does not work for other gambling patrons.

## Contributions to Theory and Practice, Limitations and Further Research

At a general level, our research finds that the COVID-19 restrictions have had negative impacts on individuals’ relational strength, and that this has negatively affected mental health and outlooks of the post-pandemic future. By focusing on casino patrons in Australia, we find that one method that they use to help cope with the negative impacts of pandemic restrictions as well as their pre-existing addictive behaviors is by increasing their alcohol consumption. Thus, alcohol consumption served as a substitute for casino gambling during the lockdown period. The results also suggest that increase of alcohol use was negatively related to life satisfaction. Moreover, the model in Study 2 is only relevant for the casino patron group, we did not find such relationships among other gambling patrons, who appear to be less affected by the pandemic restrictions. This paper holds a number of implications for theory and practice.

### Theoretical Implications from Study 1

Study 1 contributes to understanding the importance of social networks and mental health among consumers in the hospitality industry by examining the COVID-19 impact on individuals’ relational strength of their bonding relationships and its secondary impacts on their mental health and future expectations. In studying casino patrons, we believe that this is one of the few studies in hospitality management research that applies relational strength perspectives in the pandemic context, and it therefore provides some helpful insights into the relationship between social networks and mental health in a time of a major global crisis.

Our analysis suggests that when individuals’ relational strength, i.e. one’s accessibility and quality of relationships with his/her family members and close friends, is confined by major external restrictions such as the pandemic lockdown, their mental health is likely to be negatively affected. The results support other existing studies which suggest that lower level of social capital/social network strength are associated with higher risk of mental distress and psychological well-being (De Silva et al., 2007; Economou et al., 2014; Phongsavan et al., 2006). In terms of hospitality research, previous studies have corroborated the importance of enhancing social relationships from hospitality firms (Dai et al., 2015) and employees’ (King & Lee, 2016) perspectives, whereas research among hospitality customers has been limited. This study addresses this gap and highlights the critical role played by social networks among gambling patrons.

This study also offers some support to separation anxiety theory (Bögels et al., 2013). Separation anxiety was originally regarded as a “childhood onset” disorder in psychology research (Bowlby, 1960), whereas it has gained increasing attention among adults (Bögels et al., 2013). This study, while not a clinical psychology investigation, confirms a close correlation between the negative impact of separated close-knit relationships by the pandemic restrictions (i.e. relational strength) and anxiety. In light of the results, we argue that future studies on separation anxiety could delve more into the pandemic and lockdown settings in the hospitality sector.

An interesting finding is that relational strength impact has a more negative direct impact on people’s post-disaster outlook than on their current life satisfaction. While the finding is consistent across the three groups, it is most significant among casino patrons. The existing literature on social networks and mental health relations is mostly focused on individuals’ present mental health conditions, and we suggest that more studies are needed for customers in the hospitality industry to shed light on individuals’ future expectations.

### Theoretical Implications from Study 2

Study 2 contributes to understanding SRD better by offering empirical evidence in a pandemic context. The main tenets of SRD are that some individuals use alcohol drinking to cope with external stressors (Levenson et al., 1980; Sher & Levenson, 1982). The ongoing pandemic is distinct from other stressors that have been examined in previous SRD studies, therefore, this study bears several implications for the SRD and addictive behavior literature as follows:

First, as many previous SRD studies have suggested, certain individuals consume alcohol when the external stress is high. In this study, only 25.0% of casino patrons reported a decrease in alcohol consumption since the outbreak. Second, individuals’ previous alcohol consumption dependency has a strong predictive power on DTC when under stress in both gambling patron groups (β = 0.41 & 0.36; *p* < 0.01). This supports SRD’s arguments that individuals’ alcohol consumption habits in normal times is an important predictor of their DTC when under stress (Sher & Levenson, 1982; Sher & Walitzer, 1986). Third, individuals’ gambling dependency also can predict their DTC. However, this relationship was only significant in the casino patrons group and not among non-casino gambling patrons. This reinforces the finding that during the pandemic lockdown period, casino patrons tended to substitute gambling with increased alcohol consumption as a means of DTC due to the closure of gambling venues. Non-casino gambling patrons, on the contrary, were less affected by the pandemic restrictions likely because they could still access their gambling outlets during the lockdown. Fourth, when the external stressor is extraordinary, long-lasting and uncertain like COVID-19, and if the individual is highly dependent on addictive behaviors, then alcohol drinking as a stress-coping strategy, could cause negative health and subsequent social effects. The results can help to untangle the ambiguity in the SRD literature regarding the consequences of DTC—whether it can alleviate stress or not. In our study, the casino patrons group had the highest prevalence of alcohol drinking, and thus the negative correlation between the increase of alcohol consumption and life satisfaction was identified among casino patrons but not among the other non-casino gambling patrons. Fifth, although relational strength moderated the effects of DTC on life satisfaction among casino patrons, it did not do so among non-casino gambling patrons. This means the negative effect of increase in alcohol drinking on life satisfaction was stronger in those casino patrons whose relational strength was more negatively affected by the pandemic restrictions. The results imply that casino gambling activities may involve more interpersonal interactions and social networking behaviors than other gambling outlets. Lastly, there is a noticeable gender difference in the effects of DTC, where the negative impact of alcohol consumption was found to be significant in male casino patrons cohort but not significant among female casino patrons. This supports research in other gender comparison-focused SRD studies (Sinha et al., 1998).

### Managerial and Policy Implications

For the heavily repeat-customers-reliant gambling industry (Prentice, 2013) and gambling-reliant Australian tax-funded governments (AGC, 2019), it has never been more important to understand gambling patrons’ attitudes and behaviors during this unprecedented pandemic. At the time of writing, some gambling venues in Australia are preparing to resume operations (Asher, 2020). Therefore, this study provides some implications on how the casinos can regain their lost patrons in the post-pandemic era and sustain a healthy and sustainable customer relationship in the long run.

First, casinos and other gambling venues should consider implementing and strengthening product and customer diversification strategies. Moderate product diversification can improve casinos’ performance (Kang et al., 2011) as our research has shown. While casinos are struggling to survive the pandemic, there may be a silver lining for liquor retailers, another player in the hospitality sector, as casino patrons in this study were found to substitute gambling with alcohol consumption during the lockdown. In the Australian context, many casino and gambling operators also operate liquor stores and similar licenses. Therefore, even if the casino operations were closed during the lockdown, which was exacerbated by a dramatic drop in international and even out-of-state casino patrons since the outbreak (Forbes & Dyer, 2020), the liquor retailing businesses were still able to generate income to offset some of the losses from the gambling operations, and even increase them through novel business models such as online and take-away alcohol sales, which have contributed to an estimated jump of more than 30 percent of year-over-year growth in alcohol retail sales for the April-June 2020 quarter (Colbert et al., 2020).

Second, policymakers and authorities should be prudent that although gambling may have declined, the increased alcohol consumption may lead to similar significant health and social problems. For example, recent preliminary studies have found an increase in alcohol consumption and alcohol misuse among the general population during the pandemic (Clay & Parker, 2020; Da et al., 2020) and this has led to a major spike in domestic violence, some of which is alcohol-fueled (Ramalho, 2020). Of particular concern is that over one-fifth of Australia’s gambling patrons have been identified as “problem gamblers” (Armstrong & Carroll, 2017). Hence, after a long period of lockdown which has had negative effects on mental health, there may be a substantial damaging bounce-back as individuals make up by excessive gambling, which could eliminate any potential savings they may have made during the restrictions. The problem gamblers may also continue life at a higher level of alcohol consumption. This may explain a dramatic rise in alcohol-related crime in regions that have eased restrictions due to lower rates of COVID-19 infections (McNeill, 2020).

Third, and related to the second point above, governments and gambling or alcohol addiction support groups need to be especially attentive to the mental health of these individuals to mitigate against this undesirable side-effect as a result of COVID-19 restrictions. While physical distancing and staying at home are key steps to slow the spread of coronavirus, substance abuse and addiction support groups are seeing a rise in people who use or are experiencing a dependence on alcohol and other drugs, and have additional challenges and harms as a result of these measures (Alcohol & Drug Foundation, 2020). While some initial steps have been taken, the hospitality industry should work together with the government and these support groups to develop policies and measures to help these individuals avoid plunging into the vicious circle of gambling, alcohol consumption, violence and/or depression.

### Limitations and Future Studies

This study also has some limitations which can be addressed in future studies. First, despite the advantages of using a probability-based nationally representative survey, the secondary data has restricted us from developing further variables to gain a more comprehensive understanding of the research questions. Second, this study only focuses on the bonding networks of individuals’ social capital, whereas the bridging relationships may also play an important role, and future studies can attempt to shed light on this. Third, the sample size is limited, especially the casino patrons group, the significance of parameter estimates could have been compromised by the effect of the sample size. Last, our study is confined to the Australian context, and for more generalisable conclusions, it should be extended to other countries with different cultural backgrounds, different legal systems, and most importantly, different pandemic severities. This would also include testing the effect of differences in age and education in other samples as they did not seem to have an effect in our study.

## Data Availability

The data that support the findings of this study are available from the corresponding author upon request.
